# Sex and gender in infection and immunity: addressing the bottlenecks from basic science to public health and clinical applications

**DOI:** 10.1098/rsos.221628

**Published:** 2023-07-05

**Authors:** Chloé Pasin, Camila R. Consiglio, Jana S. Huisman, Ann-Marie G. de Lange, Hannah Peckham, Enriqueta Vallejo-Yagüe, Irene A. Abela, Ulrika Islander, Nadia Neuner-Jehle, Maria Pujantell, Olivia Roth, Melanie Schirmer, Burcu Tepekule, Marius Zeeb, Anna Hachfeld, Karoline Aebi-Popp, Roger D. Kouyos, Sebastian Bonhoeffer

**Affiliations:** ^1^ Collegium Helveticum, 8092 Zurich, Switzerland; ^2^ Institute of Medical Virology, University of Zurich, 8057 Zurich, Switzerland; ^3^ Department of Infectious Diseases and Hospital Epidemiology, University Hospital Zurich, University of Zurich, 8091 Zurich, Switzerland; ^4^ Department of Women's and Children's Health, Karolinska Institutet, 17165 Stockholm, Sweden; ^5^ Institute of Integrative Biology, ETH Zurich, 8092 Zurich, Switzerland; ^6^ Physics of Living Systems, Massachusetts Institute of Technology, Cambridge, MA 02139, USA; ^7^ Department of Clinical Neurosciences, Lausanne University Hospital (CHUV) and University of Lausanne, 1011 Lausanne, Switzerland; ^8^ Department of Psychology, University of Oslo, 0373 Oslo, Norway; ^9^ Department of Psychiatry, University of Oxford, Oxford OX3 7JX, UK; ^10^ Centre for Adolescent Rheumatology Versus Arthritis at UCL, UCLH and GOSH, London WC1E 6JF, UK; ^11^ Institute of Pharmaceutical Sciences, ETH Zurich, 8093 Zurich, Switzerland; ^12^ Department of Rheumatology and Inflammation Research, University of Gothenburg, 40530 Gothenburg, Sweden; ^13^ SciLifeLab, University of Gothenburg, 40530 Gothenburg, Sweden; ^14^ Institute of Immunology, University Medical Center Hamburg-Eppendorf, 20251 Hamburg, Germany; ^15^ Leibniz Institute of Virology, 20251 Hamburg, Germany; ^16^ Marine Evolutionary Biology, Zoological Institute, Christian-Albrechts-University Kiel, 24118 Kiel, Germany; ^17^ Emmy Noether Group for Computational Microbiome Research, ZIEL – Institute for Food and Health, Technical University of Munich, 85354 Freising, Germany; ^18^ Department of Infectious Diseases, University Hospital and University of Bern, 3012 Bern, Switzerland; ^19^ Department of Obstetrics and Gynecology, Lindenhofspital, 3012 Bern, Switzerland

**Keywords:** sex and gender, infection, immunity, bottlenecks

## Abstract

Although sex and gender are recognized as major determinants of health and immunity, their role is rarely considered in clinical practice and public health. We identified six bottlenecks preventing the inclusion of sex and gender considerations from basic science to clinical practice, precision medicine and public health policies. (i) A terminology-related bottleneck, linked to the definitions of sex and gender themselves, and the lack of consensus on how to evaluate gender. (ii) A data-related bottleneck, due to gaps in sex-disaggregated data, data on trans/non-binary people and gender identity. (iii) A translational bottleneck, limited by animal models and the underrepresentation of gender minorities in biomedical studies. (iv) A statistical bottleneck, with inappropriate statistical analyses and results interpretation. (v) An ethical bottleneck posed by the underrepresentation of pregnant people and gender minorities in clinical studies. (vi) A structural bottleneck, as systemic bias and discriminations affect not only academic research but also decision makers. We specify guidelines for researchers, scientific journals, funding agencies and academic institutions to address these bottlenecks. Following such guidelines will support the development of more efficient and equitable care strategies for all.

## 1. Introduction

Sex as a biological factor and gender as a social norm are recognized to be major determinants of health and human immune variation [1–8]. This results in sex and gender disparities in the prevalence and course of autoimmune diseases [9,10], cancer [11], asthma and atopy [12], neurodegenerative diseases [13] and infectious diseases—including COVID-19 [14–16] and long COVID [17,18]. Sex differences in infection outcomes have been observed in many vertebrate animals [19], suggesting that these differences have been shaped by evolutionary processes [20,21]. However, there is a remarkable discrepancy between the general but well-supported knowledge that sex and gender play an important role in infection and immunity, and the rarity with which this role is considered in clinical practice and public health. For example, most COVID-19 vaccination policies implemented worldwide were sex- and gender-blind [22], i.e. ignoring biological sex as well as gender-related factors. In addition, pregnant people were not included in the first phase III clinical trials on SARS-CoV-2 mRNA vaccines [23]. This limited the availability of data on vaccine safety and immunogenicity in this group and the subsequent vaccine coverage, despite the evidence that pregnancy may increase the risk of severe viral infection [24–26].

The disregard for sex and gender in clinical research, practice and policy may partly result from the historical belief that male anatomy, clinical signs and symptoms represent the norm [27–29]. Furthermore, women have also historically been excluded and underrepresented in clinical trials and biomedical studies [30,31]. This has been detrimental for women's health and continues to be a problem [32], as women are more frequently misdiagnosed and experience disproportionately more adverse drug effect events than men [31]. Furthermore, lack of clinical knowledge and studies on health outcomes in transgender, non-binary and intersex people has resulted in significant health disparities and barriers to care access for these minoritized groups [33,34].

**Figure 1.**
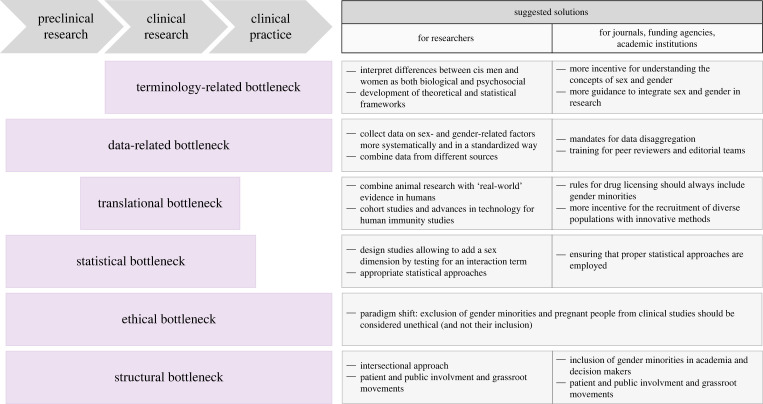
Bottlenecks preventing the integration of sex and gender into immunity research from basic science to clinical practice. This figure shows research levels affected by each bottleneck, how they add up in a timeline manner, and what solutions can be implemented by researchers and by scientific journals, funding agencies and academic institutions.

As a research field with the potential to find and develop new treatments for global threats such as pandemic diseases [35], immunology should be upfront about sex- and gender-related questions. Increased efforts from researchers, scientific journals, funding agencies and academic institutions are required to integrate sex and gender fully into infection and immunity research and translate the resulting insights into clinical practice. In this article, we discuss six major bottlenecks preventing these efforts and suggest potential solutions to address them (figure 1). The order in which the bottlenecks are described does not reflect their importance.

### 1.1. Terminology-related

**Figure 2.**
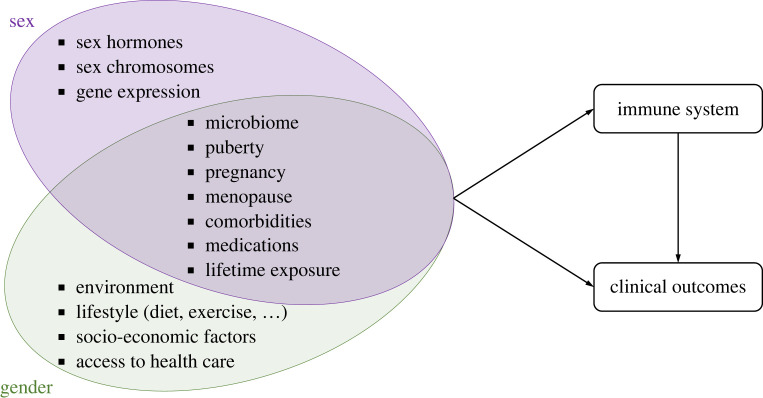
Main factors underlying sex and gender differences in immunity. These differences can be attributed to diverse causal pathways that we have classified into those mostly biologically driven (sex chromosomes, hormones and gene expression), those mostly related to environment and social factors (lifestyle, socioeconomic factors and access to healthcare) and those at the intersection of both biological and social domains (microbiome, puberty, pregnancy, menopause, medications, comorbidities and lifetime exposure).

In human research, the definitions of sex and gender themselves limit our ability to assess the impact of sex and gender on immunological and clinical outcomes [36]. Most biomedical research is conducted on cisgender individuals whose gender identity matches their sex recorded at birth, making it difficult to disentangle sex- and gender-related effects on health. As a result, the terms ‘sex’ and ‘gender’ are sometimes interchanged and misused in scientific publications [37], leading to confusion about these concepts. Further, there is no consensus on quantitative measures of gender and this variable is often wrongly considered as only binary for the sake of simplicity [38,39]. In addition, as gender has strong social and cultural components, it is hard to define markers of gender identity and roles that will be comparable across cultures, classes and/or countries. Sex and gender descriptions are only proxies for many other variables, including social and cultural components. Mechanisms underlying sex and gender differences (figure 2) need to be unravelled [40,41] to make findings translatable to public health and clinical practice.

Scientific journals, funding agencies and academic institutions should provide stronger incentives for researchers to reach a general understanding of the concepts of sex and gender. Differences observed between cisgender men and cisgender women must be interpreted as a combination of both biological and psychosocial differences—and not solely sex differences. For example, there is increasing evidence that responses to influenza infection and vaccination differ by sex [42–45] and that this can be partly attributed to variations in sex hormones. However, work from different scientific fields suggests that environment- (and therefore gender-) related factors can induce variation in sex hormone levels [46,47]. Additionally, a variety of socioeconomic factors (including those associated with gender) contribute to exposure risk for influenza infection and past build-up of immunity [48]. This implies that the interpretation of observed differences between cisgender men and women in influenza infection and vaccination should include a gender dimension. To this end, more guidance should be provided to researchers for conceptualizing and integrating sex and gender in their work (e.g. how to collect data on gender) [49–51]. Theoretical and statistical conceptual frameworks should also be developed to allow identifying sex- and gender-related causal pathways [52–56]. Further research on how to integrate gender in health studies should bridge between social sciences and quantitative and biomedical research [57].

### 1.2. Data-related

Data have been mostly generated from cisgender men and gaps in sex-disaggregated data reporting still remain [58]. Further studies are needed to understand how fluctuations in sex hormone levels, such as during the menstrual cycle, pregnancy, menopause/andropause and oral contraceptive intake, affect immunity [59–63], and how these affect clinical and treatment outcomes. Data are also missing regarding health outcomes in trans/non-binary people, e.g. on short- and long-term immune effects of exogenous sex hormone intake or minority stress (resulting from the experience of stigma and discriminations) [64]. More generally, data collected in clinical trials, electronic healthcare records, disease registries or observational cohorts are often limited to ‘sex recorded at birth’, limiting the ability to integrate gender-related aspects into data analysis. Even with recent incentives to report sex in biomedical studies [65], including the Sex and Gender Equity in Research (SAGER) guidelines which have been endorsed by journals, publishers and funders [66], there are still gaps in sex-related result reporting [58,67–70]. Confusion also arises from reporting guidelines themselves when the concepts of sex and gender are not correctly used [71].

Sex and gender need to be increasingly considered to ensure rigorous, reproducible, equitable and inclusive research [72,73]. To address this bottleneck, we believe that scientific journals, funding agencies and academic institutions should improve their guidelines and policies [74] and continue to encourage researchers to include sex- and gender-related dimensions in their work [75–83]. To this end, others have argued for mandates from funders and editors for data disaggregation, checkpoints in the submission process for ensuring that a gender perspective is included in the research work, and training for peer reviewers and editorial teams [78]. In addition, human studies should at least collect information on gender identity in addition to ‘sex recorded at birth’ to account for the role of socially constructed norms. Biomedical studies should only be allowed to exclude sex-, gender- and ethnicity-based data collection and analyses if this omission can be argued well. Given the difficulty to identify factors responsible for potential sex and gender differences in immunity (figure 2) and the diversity and complexity of potential causal pathways, we suggest collecting relevant data more systematically: sex hormone levels, intake of exogenous sex hormones and contraceptive methods, menstrual cycle phases, Tanner pubertal staging, parity, self-reported gender identity, behavioural and socioeconomic variables (e.g. care burden), sexual history and behaviour, body mass index (BMI), epigenetic age, microbiome composition, inflammatory markers. For example, sex differences in microbial composition have been observed and suggested to affect endocrinologic, metabolomic and inflammatory characteristics in the host [84]. Collecting this information would have important implications for study design, duration and funding. However, we believe that a systemic approach is necessary for unravelling the complex mechanisms underlying human immune variations related to sex and gender. Reporting on the effect of these variables in studies would facilitate building stronger evidence, for example in potential future meta-analyses. Eventually, standardized reporting on an established set of variables could be recommended to allow comparison across studies. Although this could be done without much effort for some variables (e.g. BMI, contraceptive method), more work is required on the collection and standardization of other variables, such as social determinants of health [85,86] and microbial composition [87]. Standardization of measures of sexual orientation, gender identity and sex characteristics might also be challenging from a global perspective [51]. Finally, efforts need to be made to obtain and combine data from different sources, such as real-world data (e.g. electronic healthcare records, disease registries and observational cohorts) and clinical trials data, to increase data availability on women and gender minorities.

### 1.3. Translational

Reproducibility and generalizability of basic science experiments remain a challenge, which hampers their translation to clinical research [88,89]. One particular issue is that several animal models have only considered male animals, making it difficult to translate findings to all humans [90]. Sex differences in animal models might also be difficult to translate to humans due to the importance of social factors or baseline immunity [91,92]. The underrepresentation of female participants in human clinical studies [93] further impedes the translation of research findings. Similarly, it is difficult to recruit individuals from minoritized populations as they have historically experienced and still experience discrimination from the medical field [94].

To address these issues, implementing complementary approaches that combine animal research with ‘real-world’ evidence in humans is essential to advancing our knowledge [95,96]. For example, recent work on the age-dependent impact of sex and gender on influenza vaccine response through sex hormone levels has shown the benefit of considering data from humans and mouse models together [44]. Indeed, mouse models can help unravelling causal pathways of host responses, and differences in mouse models versus human observations suggest species-specific results or the influence of pre-existing immunity [44]. Further work in animal models and humans could also give insights on the use of exogenous sex hormones to improve vaccine responses (e.g. in postmenopausal women) [97,98]. Additionally, animal studies could provide clues on how to disentangle sex and gender contributions in humans. Some animal work has already helped in distinguishing the effect of sex chromosomes and sex hormones on immunity [99], and the identification of factors affecting immunity but not related to sex chromosomes (e.g. parental investment [100,101]) might point toward important factors for studying gender in human studies. In clinical studies, clear rules regarding the inclusion of women and the collection of sex and gender information should be emphasized. Not only clinical trials should aim for recruiting more diverse populations [102,103], we also suggest that rules and regulations for drug licensing need to be reconsidered: we propose that gender minorities should always be included in clinical trials and considered as specific subgroups [104]. Although this would affect short-term costs, others have argued that they would be outweighed by the long-term benefits of the inclusion of diverse groups in clinical research [105]. As the recruitment of trans/non-binary people might be difficult in some settings, innovative methods should be employed to engage gender minorities in clinical trials [106], with professionals committed to ensuring these participants are treated with respect and dignity. Guidelines have also been suggested for improving the inclusion of gender minorities in clinical research, such as adjusting clinical protocols and adopting flexible approaches for recruitment and data collection, providing training to personnel involved and involving members of the transgender and non-binary communities in the research to establish trust [107–109]. Finally, as data collection in clinical trial settings might be limited, more observational studies should be conducted and include gender minorities. Indeed, by collecting longitudinal demographic, clinical, behavioural and immunological information on many participants, observational cohorts could actively address the questions around the role of socioeconomic factors and longitudinal exposure on immunity in humans. Thanks to recent advances in technology and computational methods, which are facilitating high-throughput data generation and analysis (e.g. single-cell technology, whole tissue analysis) in large-scale human studies, these immuno-epidemiological studies could enhance our ability to translate important results to human immunology [110–115].

### 1.4. Statistical

In animal studies, one common argument against the inclusion of both sexes is the difficulty to comply with the ‘3R’ principles (reduction, refinement and replacement) [116] while still having statistical power to detect treatment effect. Generally, sex and/or gender are only adjusted for and considered as confounders of the studied outcomes [117]. In some cases, sex and gender might be variables of importance or effect modifiers and interact with the exposure variables. However, a number of studies reporting sex differences lack statistical ground to do so, as they do not estimate the interaction term between sex and the studied risk factor [118]. This has for example led to inaccurate conclusions regarding the role of sex in the immune response to COVID-19 [119,120]. In addition, most studies lack power to study the immune system in minoritized populations (e.g. gender minorities).

Previous studies have shown the inclusion of both sexes in experimental set-ups that permit testing for an interaction term (between sex and variables of interest) only requires a moderate increase in total sample size [121]. Tools are available to design studies that can conform to the 3Rs while still allowing for the exploration of sex differences [121]. Well-founded and robust statistical approaches are critical, as separate analyses of treatment effects in males and females do not allow for any conclusion on sex differences unless the effects are statistically compared to each other [122]. For example, the inclusion of interaction terms in statistical models has allowed estimating potential sex-specific contributions of host factors (age, frailty, BMI) on the immune response to influenza vaccines [123]. In human studies, power analyses can be used to determine appropriate sample sizes for the different groups that may be included in the analysis (e.g. pregnant individuals and/or gender minorities). As we suggested that gender minorities should always be included in clinical trials, even with low sample sizes, innovative statistical tools and study design such as ‘N-of-1’ trials [124] should be considered to facilitate the implementation of this framework. The inclusion of more diverse gender identities in immunological research will provide valuable opportunities to investigate whether effects differ by sex, gender or both, and will be beneficial for science and health far beyond gender minority subgroups [125].

### 1.5. Ethical

Women and gender minorities are still underrepresented in clinical studies. This constitutes an ethical problem, as it has resulted in harmful drug dosing or delayed diagnosis in women. Pregnancy constitutes a particular case of the ‘perpetuated cycle of exclusion’ (from vaccine trials, research and generation of evidence, and vaccine delivery programmes) [126] which has also resulted in harmful drug dosing during pregnancies. By excluding women, gender minorities and pregnant people from research, opportunities for drug development are missed. For example, vaccines can protect pregnant people, fetuses and newborns. Including pregnant people in vaccine clinical trials would aid in the development of safe and effective vaccines for all. Similarly, studying and identifying how pregnancy and exogenous sex hormone intake during menopause potentially modulate susceptibility to autoimmune diseases such as systemic lupus erythematosus or rheumatoid arthritis [127,128] could be used for helping drug development against autoimmune diseases. Indeed, oestrogen and selective oestrogen receptor modulator are being considered as potential therapeutic options for psoriasis [129] or rheumatoid arthritis [130,131], as most patients show improvement of psoriasis during pregnancy, and as hormone replacement therapy can help decreasing inflammation and protecting against joint erosion in postmenopausal rheumatoid arthritis patients [132].

Many researchers and organizations have called for the inclusion of pregnant people in biomedical research [133–135]. The PHASES group has recommended three paradigm shifts, notably that pregnancy should be viewed as a ‘complex’ and not ‘vulnerable’ status [136]. The lack of data on drug safety and efficacy during pregnancy prevents pregnant people from making decisions that balance risk and benefit [137]. Therefore, a paradigm shift is urgently needed: the starting point of ethical considerations should be whether the exclusion of pregnant people and more generally of women and gender minorities is unethical, but not whether their inclusion is unethical.

### 1.6. Structural

Academic research and policy decisions are still subject to systemic discrimination and sexism. This affects how research on sex and gender differences in health is conducted and how results from this research are translated to public health policies and clinical care [138]. Indeed, there is a gender gap in funding, as women still face challenges in the academic funding system [139], resources are disproportionately allocated to diseases affecting primarily men [140], and research projects studying gender bias are less funded [141]. In the USA, women represent 78% of the population with autoimmune diseases, but only 7% of the average annual NIH rheumatoid arthritis budget went to women-focused research [142]. In addition, research conducted by women—who show more attention to potential sex- and gender-related effects [143,144]—is considered less publishable than research conducted by men [145]. Women are also under-cited [146] and the gender gap in publishing rate has widened during the COVID-19 crisis [147].

We echo previous recommendations to include women and gender minorities at all decision-making levels, from scientific research to policy making [148]. In addition, we recommend more outreach work and inclusion of women, gender minorities and other minoritized groups in the research process [149,150]. An intersectional approach is necessary to consider the effect of other social aspects on health [151,152], including age, ethnicity and race, socioeconomic status or sexual orientation. From that perspective, more discussions on terminology, culture, healthcare system and legislation in the lens of sex and gender should occur worldwide. Additionally, patient and public involvement—research carried ‘with’ or ‘by’ patients and public members, rather than ‘on’ or ‘about’ them [153]—should be requested by funding agencies, as done for example by the Swiss National Science Foundation in some cases [154]. Doing so will allow communities to engage in meaningful and active collaborations with researchers, make informed decisions and participate in decision-making, while collaborating in grassroots movements to generate more incentive for policymakers to implement sex- and gender-related health policies.

## 2. Conclusion

While sex is slowly being included in human biomedical research, gender remains a blind area in that field. Sex and gender differences in immunity should be viewed as opportunities to develop better vaccines and treatments for everyone. Considering sex and gender in immunity and including gender-diverse populations in the research process are urgently needed. Researchers, policymakers, pharmaceutical companies, academic institutions and scientific journals all bear a responsibility in addressing the highlighted bottlenecks to ensure that future treatments and vaccine approaches will be more effective and equitable for all.

## Data Availability

This article has no additional data.
